# Severe and enduring anorexia nervosa (SE-AN): in search of a new paradigm

**DOI:** 10.1186/s40337-015-0065-z

**Published:** 2015-07-31

**Authors:** Stephen Touyz, Phillipa Hay

**Affiliations:** School of Psychology, Faculty of Science, University of Sydney, Sydney, Australia; Centre for Health Research School of Medicine, University of Western Sydney, Sydney, Australia

Anorexia nervosa is one of the earliest psychiatric illnesses to be described in the medical literature with well documented accounts made in the 19^th^ century by both Gull [1] and Lasègue [2]. They both expressed optimism about the eventual outcome of treatment. Since then, there have been many claims about successful outcomes, but all too often only reversal of the undernourished state is achieved [3]. Treatment may have minimal impact on the persistent and unrelenting ruminations pertaining to food, shape and weight which are so characteristic of those with SE-AN. Good progress has been made in treating younger patients with a shorter duration of illness using family-based approaches [4, 5] but it is those who either fail to respond, or go on to develop a severe and enduring form of the disorder (SE-AN), that have received little or no attention to date [6].

Those living with a chronic illness, especially one as debilitating as SE-AN, are entitled to dream of a better tomorrow and to feel understood not only by the medical profession but by the world at large [7]. Almost every day, we are reminded about the extra-ordinary breakthroughs being made in the fight against cancer, whilst we continue to battle over the vexed issues of the imposition of involuntary treatment and the ethics of palliative care. Patients with SE-AN can no longer be ignored for they have suffered for far too long, having to contend with an abysmal quality of life devoid of any hope of an effective treatment on the horizon. This situation is in urgent need of address especially since there has only been one randomised controlled trial to date [8] that has specifically focussed upon those with the severe and enduring form of the illness. Much more needs to be done.

The time has now arrived to take the bold step in reconceptualising illness severity in anorexia nervosa especially since there is a growing recognition that the factors that may contribute towards its onset are not necessarily the same as the ones that may perpetuate it [9]. Our current classification system (DSM-5), although an improvement over its predecessors, remains limited in its clinical utility especially when identifying the onset of illness (when treatment is most likely to be effective) and giving special recognition to those when it becomes persistent [10, 11]. We have provided a cogent argument that a clinical staging model, that is so widespread in the conceptualisation and treatment of somatic illness, be adopted in anorexia nervosa. Such a model has been applied in malignancies, cardiac failure, auto-immune disease and burns where both prognosis and treatment are informed by stage [12]. Anorexia nervosa is ideally suited for the adoption of a staging model, because unlike any other psychiatric disorder, it has clearly delineated physical biomarkers of disease progression, for example bradycardia and raised liver enzymes [13] Like so many illnesses, anorexia nervosa exists on a spectrum. Just as there is no single treatment advocated for all cancers, there should not only be one treatment for all patients with anorexia nervosa. It is clear that a 14 year old adolescent with a 3 month history of anorexia nervosa would present differently to a 40 year old woman who has battled the illness for 25 years with multiple hospital admissions and has attempted cognitive behaviour therapy several times. Those with SE-AN are more likely to have high levels of disability, to be under- or unemployed, to be receiving welfare, supported by health benefit plans and become a significant burden to family, carers and health fund providers. In fact on measures such as quality of life, those with SE-AN have been found to be equal in impairment to those with severe depressive disorder as well as schizophrenia [14]. Such factors provide a compelling argument as to why a rehabilitation model of care, not too dissimilar to the ones advocated for those with schizophrenia, needs to be considered for those with a persistent eating disorder including highly specialised acute care when the need arises.

It goes without saying that such patients with SE-AN are amongst the most challenging found in mental health care [15]. They have a markedly reduced life expectancy with the highest mortality rate of any mental illness (at 20 years the mortality rate is 20 %). Because the onset of anorexia nervosa occurs at a young age, it is unfortunately not uncommon for death to occur in young adults in their thirties with a further 5-10 % every decade thereafter [16]. They suffer from a myriad of medical complications and are frequent but often reluctant visitors to general and specialist medical facilities as well as primary care services [6].

Most patients with SE-AN are unlikely to fully recover. Some do but they are in the minority. It is therefore extremely important not to focus solely upon symptom reduction, but to also take into account a more holistic model of care. Such a’ recovery model’ needs to take cognisance of the person as a whole by improving not only quality of life, but overall general functioning, employment and access to suitable housing as well [17]. This requires that our more traditional approaches to treatment, developed for earlier stages of severity, undergo a metamorphosis to better fit the needs of those with a chronic and often unrelenting illness [18].

In a recent randomised controlled trial we attempted to capitalise on those principles advocated by the ‘recovery model’ by comparing two psychological treatments which were specifically adapted for those with a more chronic disorder (Cognitive Behaviour Therapy (CBT-SE) and Specialist Supportive Clinical Management (SSCM-SE)) [8]. The hallmark and defining feature of this study, was that for the first time symptom reduction was not designated as a primary outcome measure. Weight gain was actively promoted but the primary goal was an enhanced quality of life. Both treatment arms were successful in promoting change and by the 12^th^ month follow-up period, those patients receiving CBT-SE had lower Eating Disorders Examination global scores as well as a higher readiness to recover than those receiving SSCM-SE. However, the standout feature was the remarkably low treatment attrition rate of 13 % [19] which to our knowledge is one of the lowest ever reported. We need to rethink our treatment strategies by drawing upon the patient’s strengths and competencies rather than merely paying attention to what is ‘wrong’ with them. Undertaking treatment with a poorly motivated, chronically ill patient where loneliness, despair and an empty sense of self prevail, poses unique challenges for clinicians. As Strober [15] has cautioned, such a therapeutic endeavour requires a temperament capable of enduring hours of “…sameness, respect for solitude, the ability to face frailty and profound sickness with relative ease and the ability and willingness to explore the wounds and deprivation of a life passed by”. It is also so important to never lose the sense of hope as some (albeit few) go on to make a complete recovery.

Because of the plight of those afflicted with SE-AN [20], researchers and clinicians are now pushing the frontiers of science by exploring new and bold avenues of investigation such as deep brain stimulation [18, 21] and transcranial magnetic stimulation [18, 22]. As we delve into this brave new world, it is important not lose sight of the first rule of medicine - *primum non nocere*. Some people with SE-AN are so desperate to seek relief for their untold misery that they will agree to almost anything that promises relief from their suffering and despair. The ethical debate has already begun as to whether interventions such as deep brain stimulation is in fact offering hope to the hopeless or merely exploiting the vulnerable [23].

There is now more than ever before a compelling need to bring such new ideas and emerging data to the fore in a timely fashion so that replication of the most promising new data can occur and the ethical considerations widely debated. The wheel may have already started to turn with a plenary session debating this issue at the next Eating Disorder Research Society Meeting [24] in Sicily in September. Furthermore, this journal (*Journal of Eating Disorders*) plans to publish a special issue entirely dedicated to those patients not only with anorexia nervosa (SE-AN), but those falling within the broader spectrum of eating disorders as well. It is hoped that such a special issue focussing entirely upon those with a persistent eating disorder will not only draw attention to this long-suffering group, but also generate new avenues of exploration as well.
